# Metastatic Prostate Cancer Manifesting as Cholestatic Jaundice: A Case Report and Review of the Literature

**DOI:** 10.1155/2018/1809432

**Published:** 2018-03-26

**Authors:** Deepak Ravindranathan, Emilie Elise Hitron, Greta Anne Russler, Yue Xue, Mehmet Asim Bilen

**Affiliations:** ^1^Department of Medicine, Emory University School of Medicine, Atlanta, GA, USA; ^2^Department of Hematology and Medical Oncology, Winship Cancer Institute of Emory University, Atlanta, GA, USA; ^3^Department of Pathology, Emory University School of Medicine, Atlanta, GA, USA

## Abstract

A paraneoplastic syndrome can often present as the first manifestation of an underlying malignancy. We report a patient who presented with cholestatic jaundice as a paraneoplastic syndrome from his newly diagnosed metastatic prostate cancer. He received initial treatment with androgen deprivation therapy followed by six cycles of docetaxel resulting in resolution of his cholestatic process, normalization of liver enzyme levels, and excellent biochemical and radiographic response. To the best of our knowledge, this is the first reported case of metastatic prostate cancer with cholestatic jaundice as a paraneoplastic phenomenon to be safely treated with androgen deprivation therapy and upfront docetaxel, reflecting the latest shift in the treatment of metastatic prostate cancer.

## 1. Introduction

Paraneoplastic syndrome refers to signs and symptoms that are found in the presence of a malignancy but are not attributable to direct compression or invasion of the tumor. Several of these syndromes have been found in genitourinary malignancies. Stauffer's syndrome, first described in 1961, is a paraneoplastic syndrome found in renal cell cancer that results in hepatic dysfunction [1]. Following renal cell cancer, prostate cancer ranks as the second most common genitourinary malignancy to be associated with paraneoplastic syndromes; however, finding a paraneoplastic syndrome associated with prostate cancer is still very uncommon. In the medical literature, there are about a hundred cases of paraneoplastic syndromes found in prostate cancer manifesting as endocrinological, neurological, or dermatological conditions [2].

Cholestasis in patients suffering from malignancies can typically result from a bile duct obstruction either by the primary tumor itself, metastasis to the liver, or enlarged lymph nodes [3]. In fact, the paraneoplastic syndrome of cholestatic jaundice in patients with metastatic prostate cancer without any evidence of biliary duct obstruction or hepatic infiltration has been described in the medical literature [4]. Androgen deprivation therapy (ADT) has resulted in improvement of the cholestatic process as demonstrated by decline in bilirubin, liver enzymes, and prostate-specific antigen (PSA) levels [5–7]. However, none of those patients were treated with early chemotherapy in combination with ADT as per the latest guidelines in the treatment of metastatic prostate cancer [8].

Here, we describe the first case, to the best of our knowledge, of a patient with metastatic prostate cancer who presented with cholestatic jaundice as a paraneoplastic syndrome and was safely treated with both ADT and chemotherapy, consisting of six cycles of docetaxel, resulting in excellent response.

## 2. Case Presentation

**Figure 1:**
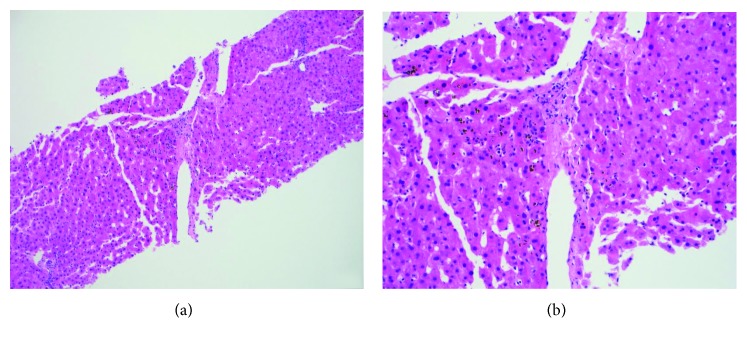
Hematoxylin-eosin staining of the liver biopsy showing canalicular cholestasis predominantly in the centrizonal areas with no obvious hepatocyte injury. (a) Magnification ×10. (b) Magnification ×20.

**Figure 2:**
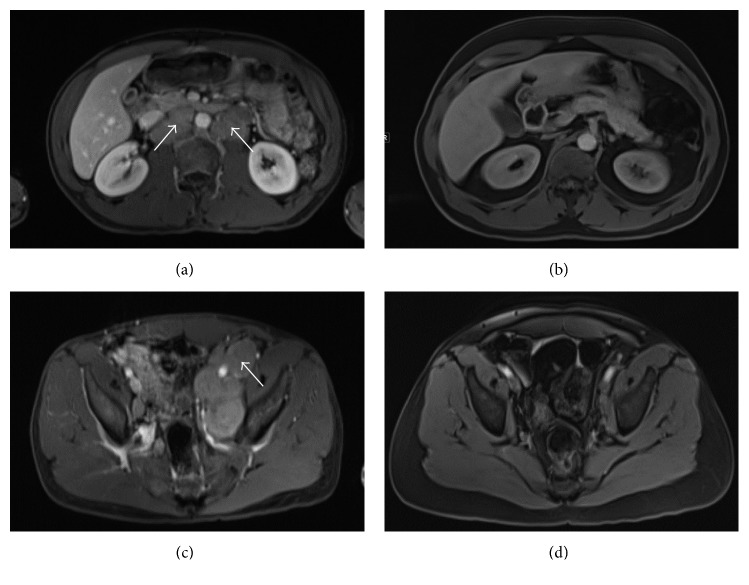
MRI of the patient's abdomen and pelvis showing resolution of paraaortic and iliac lymphadenopathy after ADT and docetaxel treatment. (a) Paraaortic lymphadenopathy (shown by white arrows) before treatment. (b) Resolution of paraaortic lymphadenopathy after treatment. (c) Left iliac lymphadenopathy (shown by white arrow) before treatment. (d) Resolution of left iliac lymphadenopathy after treatment.

A 47-year-old African American male presented at Emory University Hospital with jaundice and dark urine. He had no previous history of liver or biliary disease, blood transfusions, or recent travel. He denied fevers, night sweats, chills, nausea, vomiting, abdominal pain, distention, diarrhea, or pale stools. Initial liver function test results were as follows: AST of 425 U/L (normal range: 7–52 U/L), ALT of 601 U/L (normal range: 13–39 U/L), alkaline phosphatase of 524 U/L (normal range: 34–104 U/L), gamma-glutamyltransferase of 1389 U/L (normal range: 9–64 U/L), and total bilirubin of 15.3 mg/dL (normal range: 0.3–1 mg/dL), with direct component of 9.24 mg/dL (normal range: ≤0.18). Urinalysis showed the presence of bilirubin and urobilinogen. Viral hepatitis panel was negative. Computed tomography (CT) of the patient's abdomen and pelvis with contrast showed retroperitoneal lymphadenopathy extending into bilateral iliac chains with mottled appearance of bone, concerning metastatic malignancy. Ultrasound of the abdomen showed no evidence of cirrhosis or cholelithiasis. On endoscopic retrograde cholangiopancreatography, the cholangiogram was normal ruling out extrahepatic cholestatic disorders. Biopsy of the patient's liver showed cholestasis, mild portal chronic, and minimal lobular inflammation (Figures 1(a) and 1(b)). Magnetic resonance imaging (MRI) of the patient's abdomen revealed bulky, diffuse lymphadenopathy and secondary lymphomatous involvement of the prostate gland (Figures 2(a) and 2(b)). CT-guided lymph node biopsy revealed malignant cells, consistent with prostate cancer based on staining markers. The patient's PSA level was greater than 1300 ng/mL (normal range: ≤4 ng/mL), and his jaundice was presumed secondary to paraneoplastic syndrome from underlying metastatic prostate cancer.

**Figure 3:**
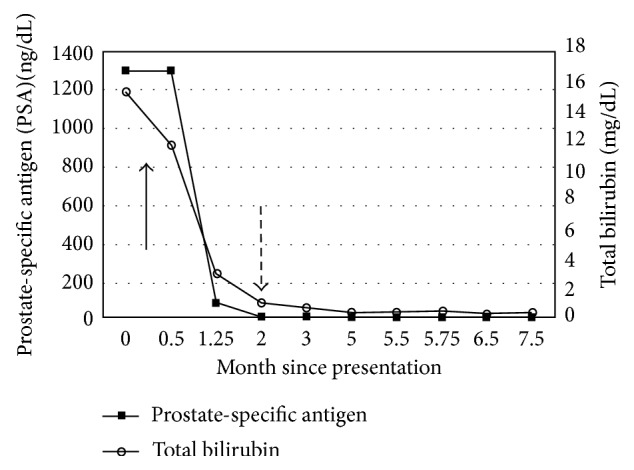
Trend in PSA and total bilirubin levels after diagnosis. Solid arrow represents the time of initiation of androgen deprivation therapy, and dotted arrow represents the time of initiation of docetaxel.

The patient was started on ADT initially with degarelix (240 mg subcutaneously) and a month later with leuprolide (22.5 mg intramuscularly every 3 months). Within 2 months of initiating ADT, his PSA level declined to 6.5 ng/mL (Figure 3). His liver enzyme levels reached normal ranges within two months, and subsequently, the first cycle of docetaxel was initiated. Patient tolerated six cycles of docetaxel without any adverse event, including no elevation in liver enzymes. The patient's most recent staging imaging after completion of chemotherapy revealed excellent response with resolution of retroperitoneal and pelvic lymphadenopathy with decreased enhancement of multiple bone lesions (Figures 2(c) and 2(d)). Notably, his most recent bilirubin was 0.3 mg/dL, and PSA was 0.84 ng/dL. At the time of writing, the patient was alive and clinically doing well.

## 3. Discussion

Timely and accurate diagnosis and treatment of paraneoplastic syndromes can greatly affect patient's clinical outcomes. Paraneoplastic syndromes may present before a cancer diagnosis; recognition of such syndrome may lead to the diagnosis of a possibly occult tumor at an early and treatable stage. The fact that this patient's jaundice was not associated with hepatocellular and cholestatic conditions and that the patient's symptoms resolved completely and shortly after initiation of antiandrogen treatment for prostate cancer suggest that the intrahepatic cholestasis in this patient was likely a paraneoplastic manifestation of prostate cancer.

The pathophysiology of this interesting paraneoplastic phenomenon is still not well delineated. Blay et al. reported that the cytokine, interleukin-6 (IL-6), is involved in the pathophysiology of paraneoplastic cholestasis in renal cell cancer. Treatment with anti-IL-6 monoclonal antibody was found to reverse most of the biochemical abnormalities in such patients. The general mechanism through which this occurs is still unknown, but this has also been thought to play a role in prostate cancer [9, 10].

There are case reports describing cholestasis as a paraneoplastic syndrome associated with several different malignancies, including Hodgkin's disease, thyroid cancer, T-cell lymphoma, chronic lymphocytic leukemia, and lung cancer [2, 11–14]. Karakolios reported a prostate cancer case that was metastatic only to the bone at the time of diagnosis and was treated with ADT, which had been the standard of care for men with metastatic prostate cancer [15]. Unfortunately, over time, patients develop castration resistance to such therapy.

Recently, there has been shift towards using upfront chemotherapy with ADT in castration-naive metastatic prostate cancer. In the CHAARTED trial, Sweeney et al. demonstrated in patients with metastatic prostate cancer that the administration of six cycles of docetaxel at the beginning of ADT treatment resulted in significantly longer survival of more than a year compared to ADT alone [16]. The STAMPEDE trial also showed survival benefit in patients with advanced prostate cancer treated with this combination [17]. Our case is the first report of a patient with metastatic prostate cancer presenting with cholestasis as a paraneoplastic syndrome to be safely treated with ADT and docetaxel. Fortunately, this treatment approach resulted in excellent response, reflecting the potential effectiveness of early chemotherapy in conjunction with ADT for advanced prostate cancer.

## 4. Conclusion

Paraneoplastic cholestasis resulting from prostate cancer should be considered in cases of jaundice in the absence of biliary or hepatic involvement or infectious etiology. We have reported such a case that was safely treated with the combination of ADT and docetaxel resulting in excellent response.
